# Supplementary figures and images for: Notch family members follow stringent requirements for intracellular domain dimerization at sequence-paired sites
Source: PLoS One. 2020 Nov 24;15(11):e0234101. doi: 10.1371/journal.pone.0234101 (PMC7685452; doi:10.1371/journal.pone.0234101)

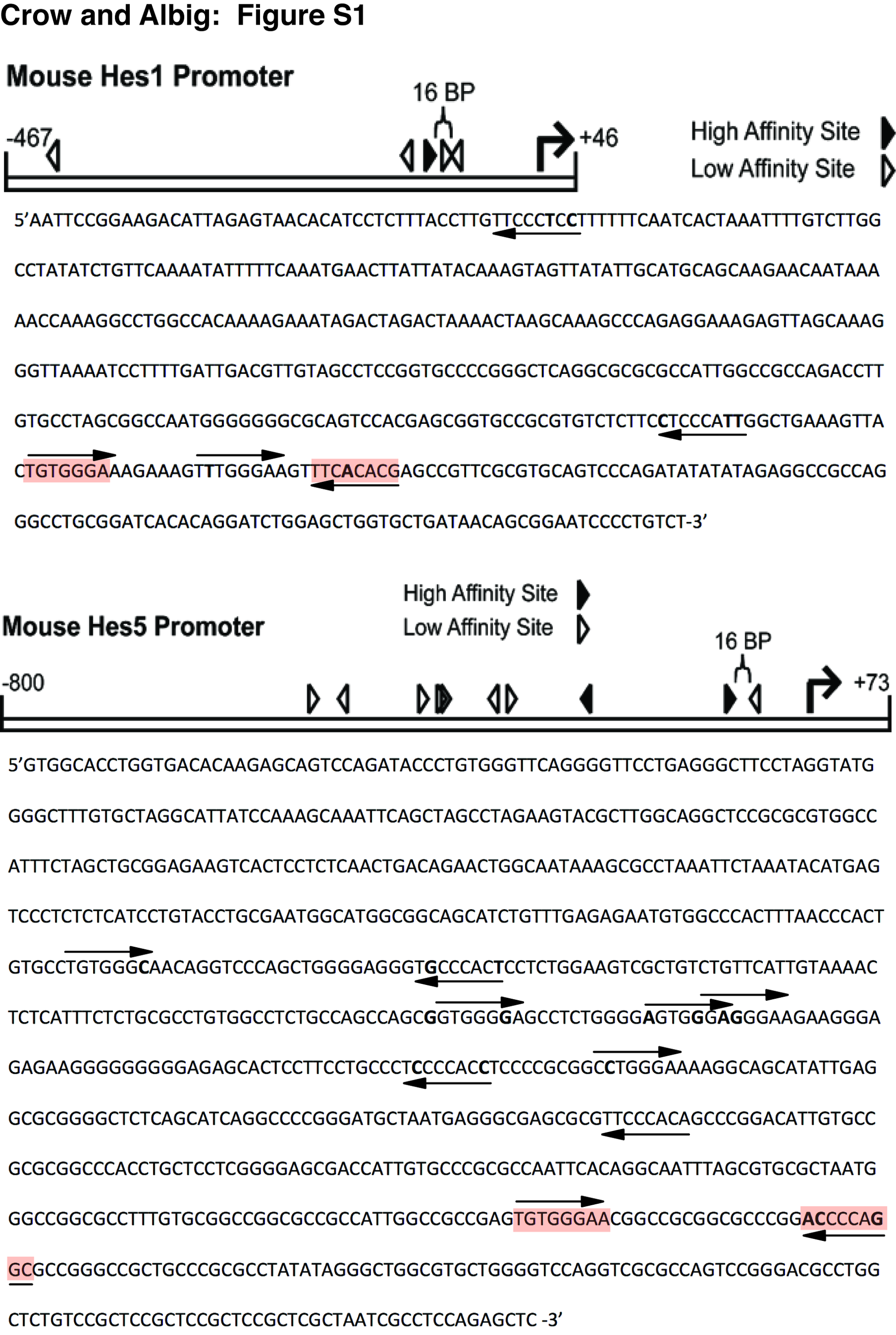

Supplement: S1 Fig — DNA sequences are as reported for Addgene products Hes1-luciferase (#41723) and Hes5-luciferase (41724) vectors. Possible high and low affinity sites (as described in material and methods) are indicated by forward or reverse arrows. High affinity sites confirmed to serve as RBPj binding sites are indicated with red shading. Nucleotides that diverge from the core TP1 element as originally defined (C/tGTGGGAA) are indicated by bolded letters. (TIF) [file pone.0234101.s001.tif]

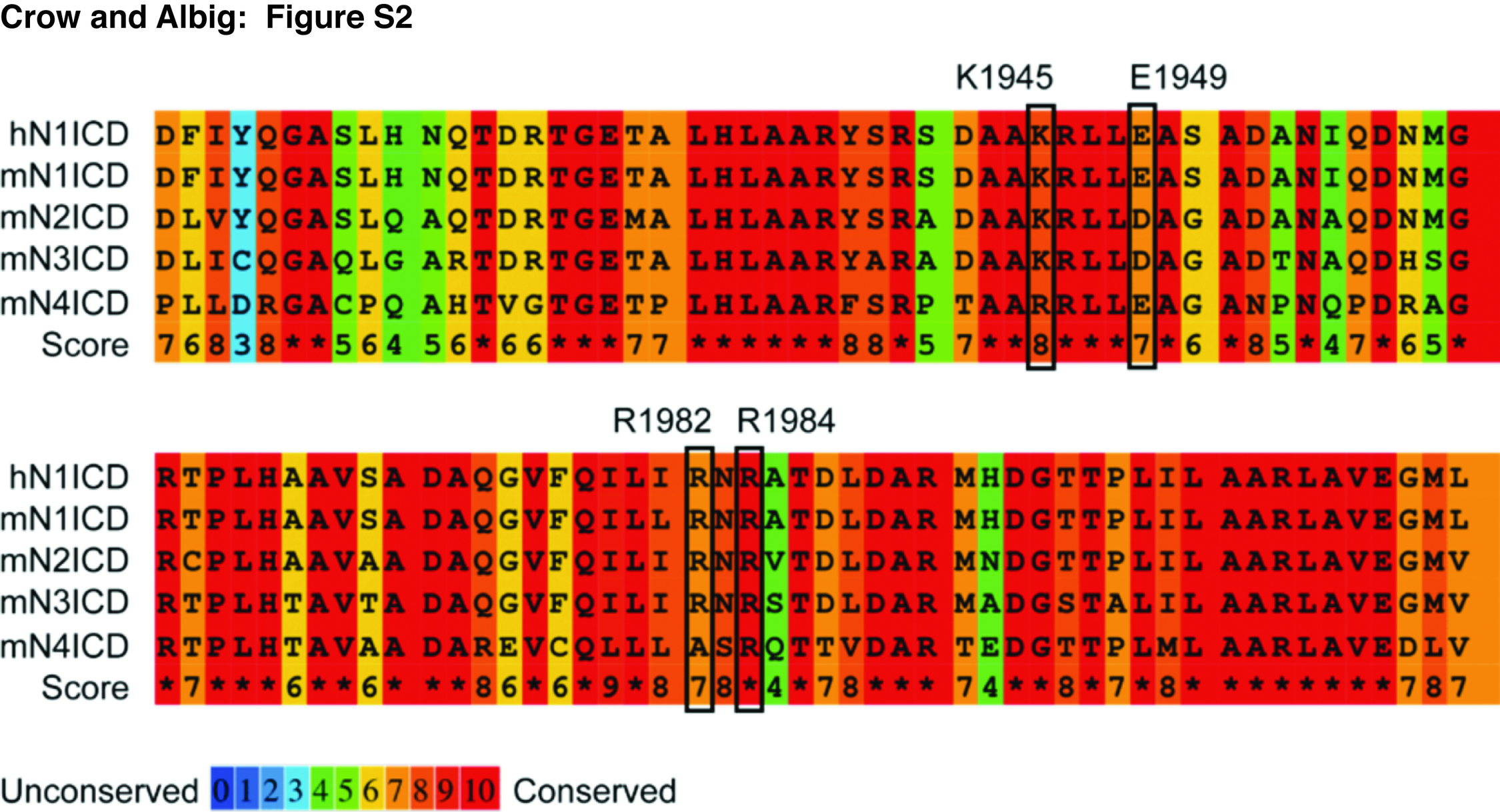

Supplement: S2 Fig — Amino acids previously identified as important for dimerization of N1ICD ankyrin domains are indicated [16]. Amino acid numbers are based on equivalent positions in human N1ICD. R1984A equivalents were used throughout this work to attempt to make generate dimer-incompetent NICD variants. (TIF) [file pone.0234101.s002.tif]

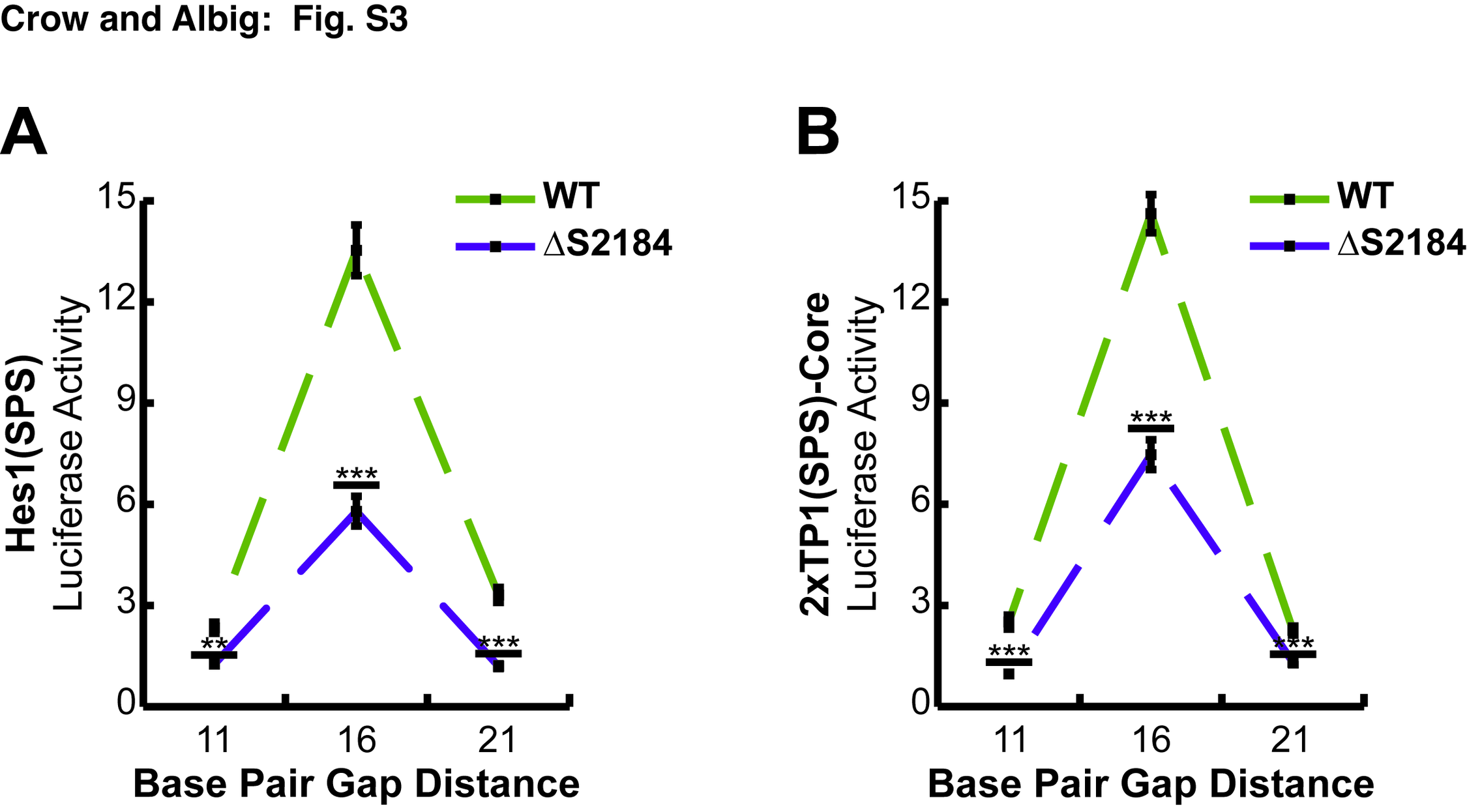

Supplement: S3 Fig — HEK293T cells were transfected with 11, 16, or 21 bp versions of Hes1(SPS) or 2xTP1(SPS)-Core luciferase reporters and either full-length N1ICD or ΔS2184 N1ICD (which deletes residues C-terminal of the N1ICD ankyrin domain). Shown is the average +/- SE of four independent experiments. (TIF) [file pone.0234101.s003.tif]

Figure 1C

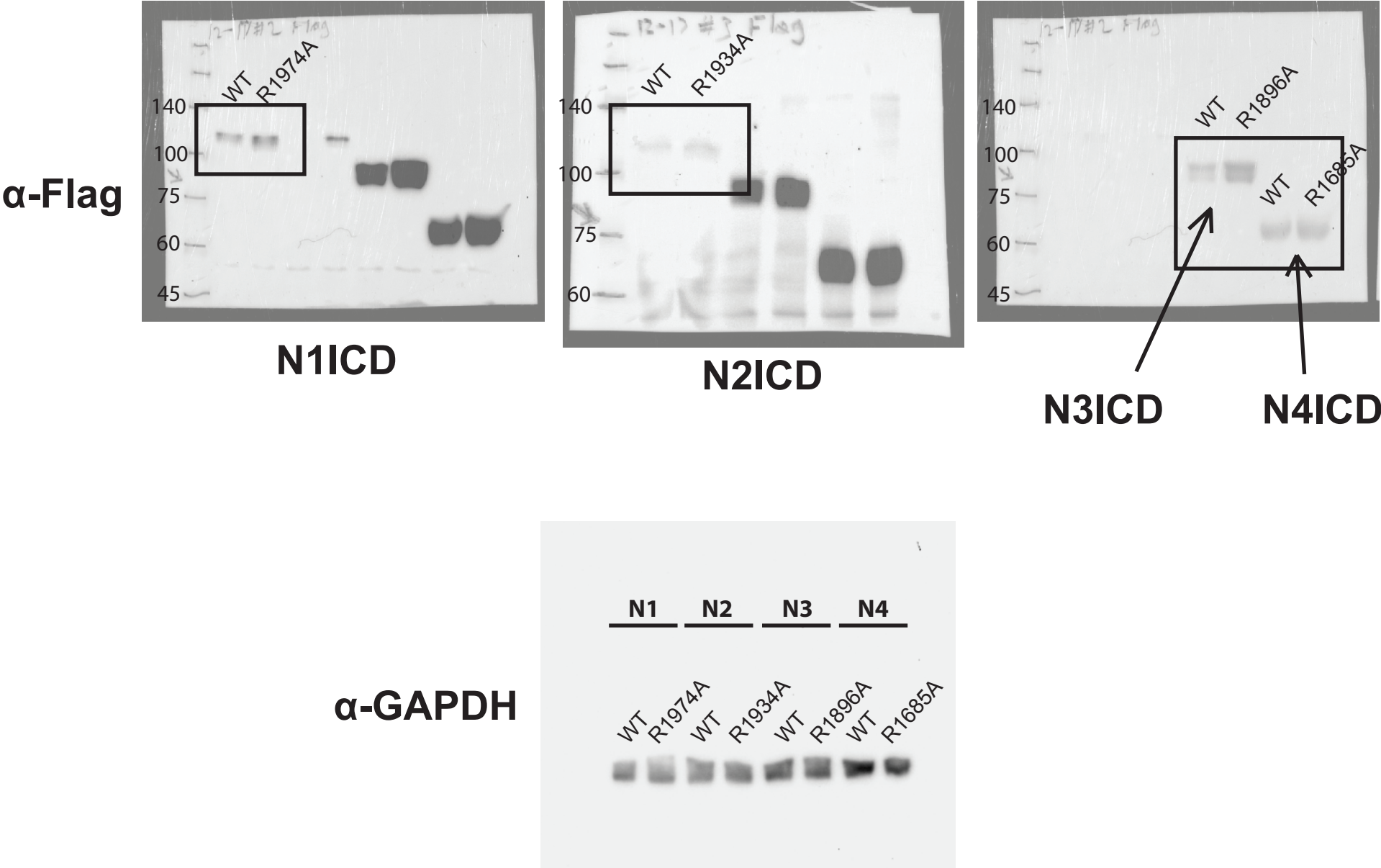

Figure 4C

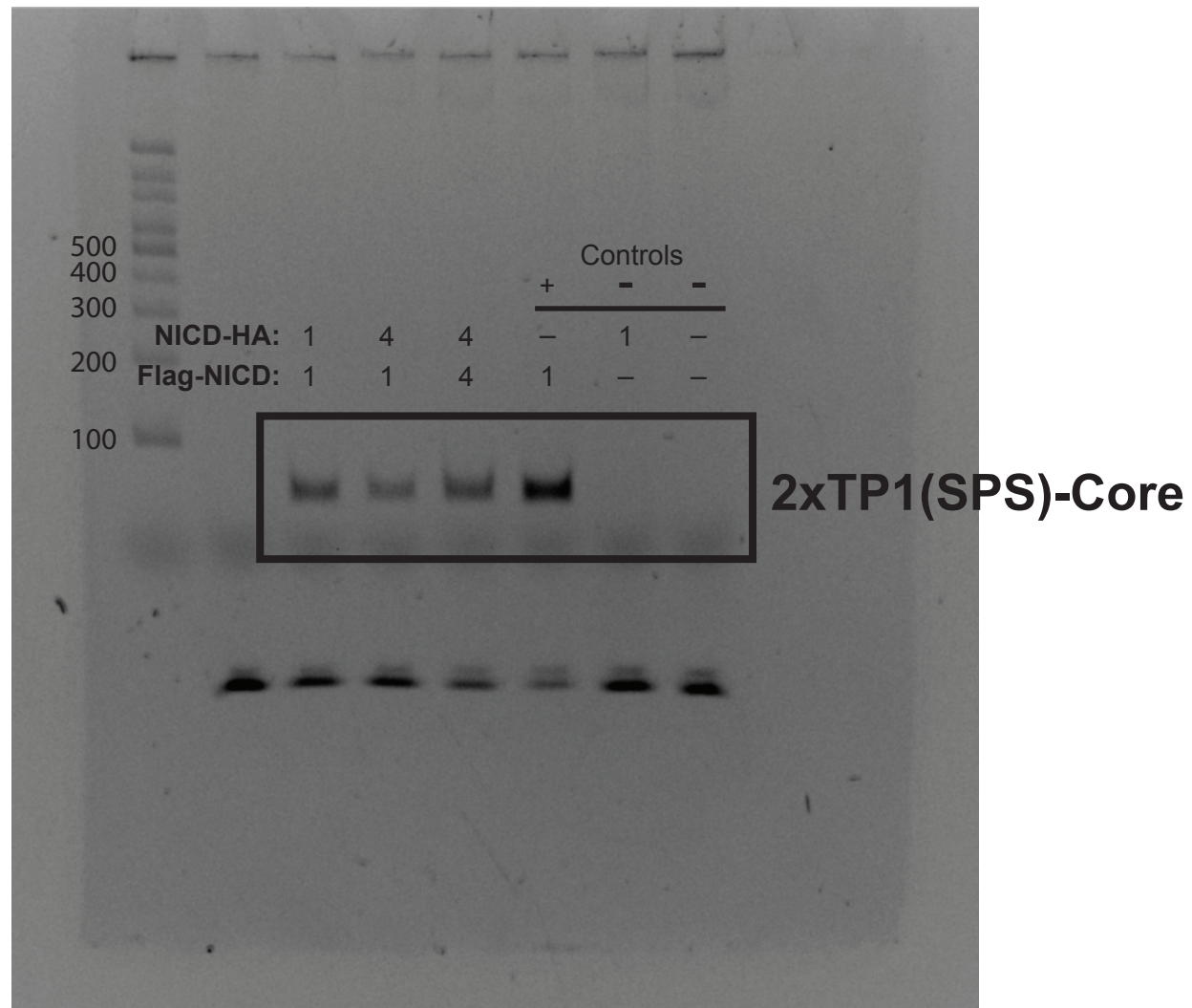

Supplement: S1 Raw images — (PDF) [file pone.0234101.s005.pdf]
