# Supplementary material for: Notch family members follow stringent requirements for intracellular domain dimerization at sequence-paired sites
Source: PLoS One. 2020 Nov 24;15(11):e0234101. doi: 10.1371/journal.pone.0234101 (PMC7685452; doi:10.1371/journal.pone.0234101)
Supplement: S1 Table — (DOCX) [file pone.0234101.s004.docx]

**S1 Table:**

**Plasmid and Sequence Information for the NICD-Activated Sequence-Paired Sites**

**Hes1**

| **Plasmid**  **Number** | **Description** | **Sequence (5’→3’)** |
| --- | --- | --- |
| 344  [AddGene #41723](http://www.addgene.org/41723/) | Full Length  (mouse sequence) | Construct contains the sequence -467 to +46 around the transcriptional start site. |
| 339 | Human/Mouse SPS Region (16 BP Gap) | GTTAC**TGTGGGAA**AGAAAGTTTGGGAAGT**TTCACACG**AGCC |
| 338 | 11 BP Gap | GTTAC**TGTGGGAA**AGAAAGTT-----AGT**TTCACACG**AGCC |
| 340 | 21 BP Gap | GTTAC**TGTGGGAA**AGAAAGTT[GGAAT]TGGGAAGT**TTCACACG**AGCC |
| 341 | 26 BP Gap | GTTAC**TGTGGGAA**AGAAAGTT[GGAAT][GGAAT]TGGGAAGT**TTCACACG**AGCC |
| 342 | 31 BP Gap | GTTAC**TGTGGGAA**AGAAAGTT[GGAAT][GGAAT][GGAAT]TGGGAAGT**TTCACACG**AGCC |
| 343 | 36 BP Gap | GTTAC**TGTGGGAA**AGAAAGTT[GGAAT][GGAAT][GGAAT][GGAAT]TGGGAAGT**TTCACACG**AGCC |

**Hes5**

| **Plasmid**  **Number** | **Description** | **Sequence (5’→3’)** |
| --- | --- | --- |
| 196  [AddGene #41724](http://www.addgene.org/41724/) | Full Length  (mouse sequence) | Construct contains the sequence -800 to +73 around the transcriptional start site. |
| 314 | Human SPS Region | CGGAG**TGTGGGAA**CGGCCGCGGCGCCCGG**ACTCCAGG**CGCC |
| 315 | Mouse SPS Region (16 BP Gap) | CCGAG**TGTGGGAA**CGGCCGCGGCGCCCGG**ACCCCAGG**CGCC |
| 317 | 11 BP Gap | CCGAG**TGTGGGAA**CGG-----GCGCCCGG**ACCCCAGG**CGCC |
| 318 | 21 BP Gap | CCGAG**TGTGGGAA**CGGCCGCG**[**GGCCC**]**GCGCCCGG**ACCCCAGG**CGCC |
| 319 | 26 BP Gap | CCGAG**TGTGGGAA**CGGCCGCG**[**GGCCC**][**GGCCC**]**GCGCCCGG**ACCCCAGG**CGCC |
| 320 | 31 BP Gap | CCGAG**TGTGGGAA**CGGCCGCG**[**GGCCC**][**GGCCC**][**GGCCC**]**GCGCCCGG**ACCCCAGG**CGCC |
| 321 | 36 BP Gap | CCGAG**TGTGGGAA**CGGCCGCG**[**GGCCC**][**GGCCC**][**GGCCC**][**GGCCC**]**GCGCCCGG**ACCCCAGG**CGCC |

**2xTP1 (Head-to-Head)**

| **Plasmid**  **Number** | **Description** | **Sequence (5’→3’)** |
| --- | --- | --- |
| 323 | Complete TP1 Site | CGACT**CGTGGGAAAAT**GGGCGGAAGGGCACGG**ATTTTCCCACG**AGTA |
| 329 | Core TP1 Site (16 BP Gap) | CGAC**TGTGGGAA**GGGCGGAAGGGCACGG**TTCCCACA**GTA |
| 324 | 11 BP Gap | CGAC**TGTGGGAA**GGGCGGAA-----CGG**TTCCCACA**GTA |
| 325 | 12 BP Gap | CGAC**TGTGGGAA**GGGCGGAA----ACGG**TTCCCACA**GTA |
| 326 | 13 BP Gap | CGAC**TGTGGGAA**GGGCGGAA---CACGG**TTCCCACA**GTA |
| 327 | 14 BP Gap | CGAC**TGTGGGAA**GGGCGGAA--GCACGG**TTCCCACA**GTA |
| 328 | 15 BP Gap | CGAC**TGTGGGAA**GGGCGGAA-GGCACGG**TTCCCACA**GTA |
| 330 | 17 BP Gap | CGAC**TGTGGGAA**GGGCGGAA**[**G**]**GGGCACGG**TTCCCACA**GTA |
| 331 | 18 BP Gap | CGAC**TGTGGGAA**GGGCGGAA**[**GA**]**GGGCACGG**TTCCCACA**GTA |
| 332 | 19 BP Gap | CGAC**TGTGGGAA**GGGCGGAA**[**GAG**]**GGGCACGG**TTCCCACA**GTA |
| 333 | 20 BP Gap | CGAC**TGTGGGAA**GGGCGGAA**[**GAGG**]**GGGCACGG**TTCCCACA**GTA |
| 334 | 21 BP Gap | CGAC**TGTGGGAA**GGGCGGAA**[**GAGGC**]**GGGCACGG**TTCCCACA**GTA |
| 335 | 26 BP Gap | CGAC**TGTGGGAA**GGGCGGAA**[**GAGGC**][**GAGGC**]**GGGCACGG**TTCCCACA**GTA |
| 336 | 31 BP Gap | CGAC**TGTGGGAA**GGGCGGAA**[**GAGGC**][**GAGGC**][**GAGGC**]**GGGCACGG**TTCCCACA**GTA |
| 337 | 36 BP Gap | CGAC**TGTGGGAA**GGGCGGAA**[**GAGGC**][**GAGGC**][**GAGGC**][**GAGGC**]**GGGCACGG**TTCCCACA**GTA |

**2xTP1 (Head-to-Tail)**

| **Plasmid**  **Number** | **Description** | **Sequence (5’→3’)** |
| --- | --- | --- |
| 261 | Two TP1 Binding Elements, Head-to-Tail Arrangement | CGACT**CGTGGGAAAAT**GGGCGGAAGGGCAC**CGTGGGAAAAT**AGTA |

**4xTP1 (Head-to-Tail)**

| **Plasmid**  **Number** | **Description** | **Sequence (5’→3’)** |
| --- | --- | --- |
| 197  [AddGene #41726](http://www.addgene.org/41726/) | Four TP1 Binding Elements, Head-to-Tail Arrangement | TTCGC**CGTGGGAAAAT**CGC**CGTGGGAAAAT**CGC**CGTGGGAAAAT**CGC**CGTGGGAAAAT**TCGA |
