# Supplementary material for: Notch family members follow stringent requirements for intracellular domain dimerization at sequence-paired sites
Source: PLoS One. 2020 Nov 24;15(11):e0234101. doi: 10.1371/journal.pone.0234101 (PMC7685452; doi:10.1371/journal.pone.0234101)
Supplement: Attachment — Submitted filename: rebuttal letter #2.pdf [file pone.0234101.s007.pdf]

BOISE STATE UNIVERSITY
Department of Biological Sciences
1910 University Drive Boise, Idaho 83725-1515
phone 208-426-3262
fax 208-426-1040
bioinfo@boisestate.edu
http://www.boisestate.edu/biology/

10-26-2020

Dear Dr. Mosialos,

Thank you for continuing to serve as editor for our submission to PLOS One. We again thank reviewer #1 for his/her thoughtful review of our manuscript. I must say that I admire reviewer #1 for the dedication shown in reviewing our manuscript. In the true sense of the peer review process, our manuscript has been greatly improved due to the reviewer's diligence. I hope our new revisions will be satisfactory. We have addressed the concerns below.

1) I still do not believe the ChIP study demonstrates that N1ICD and N4ICD form heterodimers. The luciferase 2xTP1 DNA used in this assay has two Rbpj binding sites. Thus, when the ChiP-reChiP assay is done and the authors find that both N1ICD and N4ICD are bound to the same DNA – how do they know it forms a heterodimer (which is what they claim) versus simply one site bound by N4 and the other site independently bound by N1? That is why I recommended doing additional control experiments with Rbpj binding sites separated by 21 nts – which are non- cooperative sites. In fact, the authors themselves stated in the rebuttal to my comments the following: "since the 21bp promoter can still theoretically bind to two different tagged (but undimerized) NICD molecules, we would probably still detect ChIP on the 21bp promoter." But that argument is also true of a 16bp spacer as well. I also recommended testing dimer-deficient NICD molecules in this assay – to which they stated in their rebuttal: "on the surface, this sounds like an excellent control for our experiment, however, it is necessary to remember that any two NICD molecules might be able to bind to the DNA regardless if they are dimer capable or incapable." And yet, in the manuscript, the authors state that their data supports the conclusion that N1 and N4 form heterodimers on SPS sites. In my opinion, this data shows that N4 and N1 can both bind to the same DNA that has two Rbpj binding sites at the same time. It does not show that these molecules form heterodimers.

Originally, this figure was only intended to show that the 2xTP1(core) construct was in fact able to bind NICD molecules, but it has (for the better) taken on a whole new "life of its own". The reviewer is 100% correct about the concerns with the experiment and we have performed the suggested experiments. I have included the new data in this letter. The result is very interesting and raises new questions about how Notch functions. However, as is so often the case, this new data raises more questions than it answers. And so, given that the manuscript does not hinge on this data, I have opted to remove panel 4C from the final manuscript. That said, I plan to pursue this ChiP approach to see what it can teach us (if anything) about how Notch functions. Below is the data.

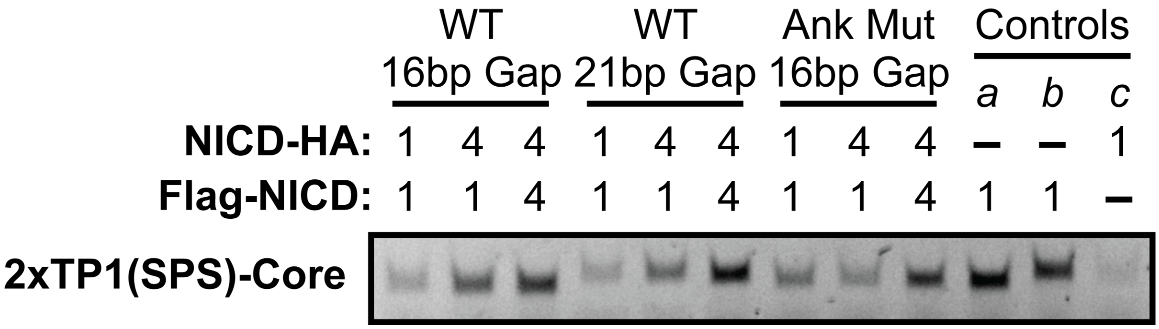

New ChiP data (not included in revised manuscript). 293T cells were transfected with various combinations of wild type (WT) or dimer null (Ank Mut) FLAG-tagged NICD, HA-tagged NICD molecules, and a reporter plasmid with RBPj binding sites orientated either 16 or 21 bp apart. NICD binding to the reporter plasmid was determined by cross-linking protein to DNA followed by a two-step ChiP method involving 1.) IP with anti-FLAG antibodies, 2.) elution with FLAG peptide, and 3.) a second IP with anti-HA antibodies. After the second IP, samples were treated with proteinase K and reverse cross-linked and subjected to PCR analysis with oligos that amplify a DNA segment centered on the plasmid RBPj binding sites (note that the 21 bp gap PCR product is slightly larger than the 16bp gap PCR product). Positive control samples "a" and "b" were transfected with the 16 (a) or 21 (b) gapped plasmids and N1ICD-FLAG then subjected to a single round of IP with anti-FLAG antibodies. Negative control sample "c" was transfected with the 16 bp gapped plasmid and N1ICD-HA then subjected to the two-step ChiP experiment as described above.

The new data shows (as the reviewer correctly guessed) that NICD molecules can associate with the DNA even if the gap between RBPj binding sites is non-optimal (21 bp) or the NICD molecules are dimer-null (Ank Mut). Keep in mind however that the N4ICD ankyrin mutation used here is the same mutation we used in the manuscript that was shown to NOT break N4ICD dimerization activity (*I have a student currently trying to solve that little N4ICD mystery*). However, the N1ICD ankyrin mutation does certainly suppress transcription from SPS sites with 16bp gaps as shown in our manuscript. Regardless, we are forced to agree with the reviewer that this data cannot distinguish between 1.) NICDs engaging in dimerization on the DNA, or 2.) two NICD molecules binding to DNA independently of dimerization.

That said, the new ChiP data does raise other questions and I would welcome the reviewers input about what this data might mean. If the reviewer feels comfortable, I would welcome a discussion in any format he/she feels appropriate with. Even as an anonymous note through the official reply to this resubmission would be welcome. In particular, the new data is particularly interesting since we consistently recover the most DNA in the 4/4 sample ChiP sample, followed by the 1/4 sample, followed by 1/1 sample. Almost always in that order. And, it is not just the transfected target DNA since ChiP analysis of the chromosomal Hes1 and Hes4 promoters returned the same pattern of DNA recovery (not shown).

If this data does not illustrate dimerization, what does it mean? Does this data suggest that N4ICD simply binds to RBPj and DNA better than N1ICD? Does it suggest an activity of NICD binding to DNA that we are not aware of? Or, maybe the data is screaming the obvious at us … maybe NICDs bind just fine to 21 bp gapped SPS

elements and they don’t need ankyrin domains to bind (this is what the data says). Maybe the only way to get the synergistic amplification of transcription that comes with NICD dimerization is to have dimerization on the 16bp gap so that MAML and p300 can bind to the a NICD complex with the correct dimensions? My student’s and I are currently scratching our heads over this one. In the end, since the data is not critical for the overall paper, I have decided to simply remove the data and save it for another time when we can more robustly analyze the experiment, perform follow up experiments, and give these questions the full attention they deserve.

2) Figure 1B needs significance tests between the WT and RA mutants for N2ICD, N3ICD, and N4ICD.

This has been updated as requested.

3) A point of clarity – In Fig 4A and 4B the sequence is provided for the SPS site tested and it is labeled TP1 (complete) and TP1 (core). However in all the luciferase data it is called 2xTP1(SPS)-complete or 2xTP1(SPS)-core. Does that mean it contains two copies of the core and complete sequence? Or should the above sequence be re-labeled as 2xTP1?

Thank you for pointing this out. These are both 2xTP1 elements and we have modified the description of each DNA sequence appropriately.

4) A minor comment for the authors to consider – but it is their choice to leave it or change it. I still personally find the small differences in luciferase activity between the WT and RA mutant NICD molecules to be overstated. Hence, I don't really understand why they make such a big deal about these small differences (luciferase assays are really sensitive and it is unclear what a less than a 2-fold difference means). Hence, I find their argument in the section on “Non-optimal SPS sites select against transcriptional activation by NICD dimers” to be less than compelling. But again, I am a firm supporter of the authors telling the story how they want to – I would just state that as a person that has studied transcription for over 25 years and performed luciferase assays throughout that time – it is really hard to determine the importance of such small changes, even when statistically different.

I completely understand your point here and I hesitate to fly in the face of experience. It is not a major point in our paper, however I think that I would like to keep this data in the paper. My reason for doing this is that there is not much known about how the NICD complexes actually bind DNA and initiate transcription. My gut instinct is telling me that this data might be able to teach us something about Notch. Hopefully, it will be useful for others.
