# Supplementary material for: Notch family members follow stringent requirements for intracellular domain dimerization at sequence-paired sites
Source: PLoS One. 2020 Nov 24;15(11):e0234101. doi: 10.1371/journal.pone.0234101 (PMC7685452; doi:10.1371/journal.pone.0234101)
Supplement: Attachment — Submitted filename: Rebuttal points.docx [file pone.0234101.s006.docx]

8-17-2020

Dear Dr. Mosialos

Thank you for serving as editor for our submission to PLOS One. We thank the reviewers for their thoughtful review of our manuscript and we were honored by the overall enthusiasm for our work. We have addressed the reviewers concerns on a point-by-point basis below. In each case, we repeat the reviewers concern in italics and our response below each point.

*Reviewer #1:* 
*1) An assumption made in the paper is that the same mutation that disrupts Notch1-ICD dimer formation will also disrupt N2, N3, and N4 dimer formation. Past publications used assays like EMSAs or FRET assays to show that Notch1-ICD forms dimers, but the authors provided no direct evidence that the mutations tested in the luciferase assays actually abolishes dimer formation for the other Notch molecules. Hence, any change in transcriptional activity is assumed to be caused by a decrease in dimer formation without any evidence it actually does cause that change. They need to provide such evidence or they need to dramatically change the way they write the paper about whether these mutations actually impact cooperative DNA binding.*

- The reviewer is concerned that the amino acid mutation that renders N1ICD dimer incompetent, may not also cause the other NICDs to be dimer incompetent. The reviewer is also concerned that reduced transcriptional activity of NICD mutants might be due to altered protein expression or stability. This is a fair concern and one that we would have loved to address with EMSA assay (as suggested). However, despite our best attempts, we have been unable to make the NICD EMSA assay work in our hands. As an alternative, we have resorted to our luciferase promoter assay to address the reviewer’s concerns. In our new data, we compared the transcriptional activity of both WT and mutated NICD molecules on a dimer-independent 4XTP1 luciferase construct. We rationalized that if WT and mutant NICD performed equivalently on this 4XTP1 that this would eliminate any concerns about protein expression, stability, or interactions with CSL and leave the only explanation for reduced activity on dimer-dependent promoters that the mutation blocks dimerization activity. Our new data in figure 1D shows that all the WT and mutant NICD molecules in fact do have equivalent activities on this dimer-independent construct suggesting that changes in transcriptional activity are not related to protein expression or stability. As has been noted elsewhere in this rebuttal letter, N4ICD is the exception since it does not appear to function in a dimer dependent manner based on mutation of the ankyrin domain. That said, our data does demonstrate what appears to be dimerization activity by ChIP and by the preference of N4ICD for a 16 bp template. Based on these observations, we do feel that N4ICD engages in dimerization, but that the amino acid mutated in our present experiments “missed the mark”. Instead, future research is going to be focused on identifying the critical amino acids in N4ICD that are critical for dimerization activities.

*2) A better description of the plasmid design of the synthetic luciferase reporters is needed. In particular, I did not understand how these plasmids work given the authors wrote the following: “We isolated the mouse and human Hes5 SPS elements containing two head-to-head orientated RBPJ binding sites separated by a 16-nucleotide gap, and the 4 to 5 surrounding nucleotides (Figure 2A) as previously described and cloned these sequences into promoterless luciferase reporter vectors.” The authors similarly stated that they used a promoterless pGL3-basic vector in the methods. If the luciferase vector does not have a promoter and the synthetic insert only has an SPS site – then how does RNA polymerase initiate transcription? Is the assumption here that the constructs initiate transcription in the complete absence of a promoter or* ***does it contain a minimal promoter that is insufficient to activate gene expression on its own****? If it completely lacks a promoter – the authors should provide a clear rationale for why it provides a good model to use to study Notch-dependent transcription, as all Notch target genes presumably have a promoter. Given this discrepancy, the relevance of the results can be called into question.*

- The reviewer is absolutely correct. The term “promoterless” is a sort of shorthand commonly used to describe a plasmid that has a minimal promoter, incapable of initiating transcription without additional enhancer elements (such as the SPS sites) used in our paper. We have clarified this throughout the manuscript.

*3) The authors claim in the abstract that “each family member is capable of dimerization-induced signaling”, but the authors provide no evidence for Notch4. The only evidence the authors show is that N4 induces optimal induction of a 2xTP1(SPS)core construct with a 16bp spacer in Figure 6. But the authors used that same luciferase reporter to test the N4 wild type and mutant protein and found no significant change in activation using the mutant N4 molecule in Figure 4B. Hence, no N4 mutant protein was shown to significantly change transcription via SPS sites or any other site. Hence, the authors do not have the data to support this claim.*

-We agree with the reviewer that our evidence for N4ICD dimerization is very minimal. Unfortunately, I believe that the amino acid mutation that seems to “break” dimerization in the other NICD molecules does not affect N4ICD in the same way. We have enhanced our previous figure 1 (now figure S2) to show an expanded sequence alignment that provides a better understanding into how the assumed dimerization domain is fairly different in N4ICD compared to the other NICDs. Regardless, we cannot say for sure that N4ICD does (or does not) dimerize. As the reviewer notes, our only evidence is a preference for the 16bp gap and the CHiP data in panel 4C wherein we detect N4ICD dimers by Co-IP and PCR. We attempted to emphasize this throughout the text but overlooked this comment in the abstract. This has been amended. This comment is also similar to one of reviewer two’s comments. We are currently performing experiments to determine which amino acids in N4ICD are necessary for dimerization.

*4) The authors also did double-selection ChIP-PCR experiments in Figure 4 to make the following claim: “Our analysis determined that both Notch1 and Notch4 homodimers cooperate on the 2xTP1(SPS)-Core construct and are therefore likely actively signaling as a duplex (Figure 4C). Importantly, we also observed Notch1-Notch4 heterodimers, though at a lesser abundance. This implies that two different Notch proteins can come together at the same promoter site and interact, possibly modulating the other’s transcriptional output.” I have several concerns about the interpretation of this experiment.*

*First, the authors stated that these homodimers bind in a “cooperative” manner. However, no proper positive or negative controls are included to demonstrate cooperativity. For example, the ChIP-PCR experiments should also be done on a region containing two high affinity binding sites that do not mediate cooperative binding (i.e. 21bp spacer) and show that there is a dramatic decrease in binding – which would be consistent with cooperativity.*

-Thank you, reviewer, for pointing this out. We were mistaken to suggest that we were interested in demonstrating cooperativity in this experiment. Instead, we were only interested in observing NICD dimerization. We have extensively rewritten this section in the revised manuscript. Demonstrating cooperativity would be extremely challenging by this approach since as we understand the assembly of the Notch tripartite complex, the synergy of the 16bp template is not associated with NICD/RBPj binding to DNA, but rather to the ability to recruit RNA polymerase. Therefore, since the 21 bp promoter can still theoretically bind to two differently tagged (but undimerized) NICD molecules, we would probably still detect ChIP on the 21 bp promoter. A better readout for cooperative function is in fact strong transcriptional activity on the 16bp template which is demonstrated in figure 6.

*Second, the authors need a negative control by testing a reporter with both binding sites mutated.*

-We also originally thought about using mutated SPS sites as a negative control in this experiment. However given the sensitivity of PCR as a readout, we were concerned that any residual binding (even on mutated sites) would be detected in our PCR analysis. Since we were also not attempting a quantitative approach to our analysis, this would have been difficult to control for. In the end, we feel that the two negative controls we did use are excellent. The most basic negative control we used was just the luciferase plasmid transfected with no NICD. The more robust negative control we used was the sample transfected with HA-tagged NICD and lacking Flag-tagged NICD, but still subjected to the two-step Co-IP procedure. We felt (and still do) that the absence of PCR signal in this more robust control provides very good evidence for the specificity and lack of background in our experiment.

*Third, it is standard practice to quantify this data using a fold enrichment over input chromatin.*

-It was never our intention (at this point anyway) to use this ChIP assay for anything more than a demonstration of another technique to detect dimerized NICD molecules on DNA.

*Fourth, the authors should also test the dimer mutations – especially to make the claim that N4/N1 heterodimers form – given that the authors have not convincingly shown that N4 even forms homodimers (see point 3 above).*

-On the surface, this sounds like an excellent control for our experiment however it is necessary to remember that any two NICD molecules might be able to bind to the DNA regardless if they are dimer capable or incapable. Therefore, this experiment would probably give false positives, suggesting dimerization even though it was not happening. As stated above, we really feel the controls we chose to use in this study are a good compromise to show specificity of the results and to avoid the potentially sticky false positives we sought to avoid.

*5) The authors made the following statement in the results section: “The results in figure 4A revealed an interesting phenomenon wherein non-dimerizing ankyrin mutant NICDs performed better than their WT counterparts on the 2xTP1(SPS)-Complete construct which had a slightly longer gap than the 16 bp preferred by NICD molecules. This observation suggested that two RBPJ binding sites gapped slightly more or less than 16 bp within a promoter might actually suppress promoter responsiveness to NICD dimer-dependent Notch signaling and favor NICD dimer-independent Notch signaling, a phenomenon which has not been previously described.” However the change was only from ~5-fold for the wild type protein to ~7.5-fold for the RA mutation. Couldn’t such a small relative change in luciferase activity between the wild type vs RA mutant protein actually be explained by differences in the levels of the NICD wild type and RA proteins? In looking at the Western blots in Figure 1C, it appears that the NICD-RA proteins are consistently higher than the Wild type protein – although no quantitation is provided. This increase in protein levels could explain why the RA protein is better on the 11bp and 21bp spacer luciferase constructs. Note, the increase in levels of the RA protein relative to wild type could be due to increased protein stability as has been recently suggested in Drosophila by Kuang et al eLife 2020. However, another recent paper (Kobia et al 2020 on bioarchives) found that the N2RA mutation did not dramatically alter N2ICD levels – and thus such changes in levels/stability could be cell type specific.*

-The reviewer is correct that this small change could be caused by properties of the mutant N1ICD other than dimerization potential. This concern however is addressed in the revised figure 1D wherein we compare WT and ankyrin mutant NICD transcriptional activity on dimer independent template 4xTP1. We found no significant difference between WT and mutant on this promoter suggesting that any difference between WT and mutant NICDs is based on dimerization potential and the synergistic transcriptional activity associated with NICD dimerization.

*6) The authors should better describe how they selected the sequences used in the different spacers. Were they selected to exclude additional TF binding sites? Some previously described SPS sequences were found to often contain a common hexamer sequence (typically something like GAAAGT, which is found in Hes1, see Posakony papers). Did they purposely avoid or include such sequences in their constructs? Did they scan the sequences to exclude the possibility of making additional low affinity Rbpj sites?*

-We have revised the section in the material and methods that describes plasmid construction to more precisely address this concern.

*7) Minor comment: Figure 6 needs statistical tests.*

-At the reviewers request we did perform ANOVA and Tukey-Kramer's HSD post-hoc test on this data. We have reported these results in the revised figure legend for figure 6.

*Reviewer #2:* 
*1. Authors shouldn't refer to "dimerization null constructs", but rather "dimerization incompetent constructs" throughout the text.*

-As suggested, we have made this change throughout the manuscript.

*2. In Fig 1D, it would be good if authors could exactly specify the high/low affinity DNA sequences identified in a Supplementary Fig.*

-As suggested, we have prepared a new more detailed figure S1 that shows the DNA sequences of all of the high/low affinity sites.

*3. In the discussion, authors hypothesize that R1945 might be relevant in N4ID for dimerization. Can authors check this? This should be fairly easy to do using their reporter system, upon site-directed mutagenesis for that position.*

-We agree with the reviewer that this is an experiment that needs to be done. At this time however, Mr. Crow is ready to leave the lab and the project has been passed onto a new student. This will be the subject of a future study.

*4. Please correct grammar in the Discussion in the sentence "This indicated
that long-range interactions between NTC complexes are unlikely TO occur"*

-done.
